# Recruitment of Rad51 and Rad52 to Short Telomeres Triggers a Mec1-Mediated Hypersensitivity to Double-Stranded DNA Breaks in Senescent Budding Yeast

**DOI:** 10.1371/journal.pone.0008224

**Published:** 2009-12-14

**Authors:** Yi-Hsuan Lin, Chia-Ching Chang, Chui-Wei Wong, Shu-Chun Teng

**Affiliations:** Department of Microbiology, College of Medicine, National Taiwan University, Taipei, Taiwan; Texas A&M University, United States of America

## Abstract

Telomere maintenance is required for chromosome stability, and telomeres are typically replicated by the action of telomerase. In both mammalian tumor and yeast cells that lack telomerase, telomeres are maintained by an alternative recombination mechanism. Here we demonstrated that the budding yeast *Saccharomyces cerevisiae* type I survivors derived from telomerase-deficient cells were hypersensitive to DNA damaging agents. Assays to track telomere lengths and drug sensitivity of telomerase-deficient cells from spore colonies to survivors suggested a correlation between telomere shortening and bleomycin sensitivity. Our genetic studies demonstrated that this sensitivity depends on Mec1, which signals checkpoint activation, leading to prolonged cell-cycle arrest in senescent budding yeasts. Moreover, we also observed that when cells equipped with short telomeres, recruitments of homologous recombination proteins, Rad51 and Rad52, were reduced at an HO-endonuclease-catalyzed double-strand break (DSB), while their associations were increased at chromosome ends. These results suggested that the sensitive phenotype may be attributed to the sequestration of repair proteins to compromised telomeres, thus limiting the repair capacity at bona fide DSB sites.

## Introduction

Telomeres are the specialized DNA-proteins complexes at the ends of eukaryotic chromosomes that allow intact ends to be distinguished from broken chromosomes, prevent chromosomes from end-to-end fusion, degradation, genomic instability and the associated risk of cancer. In most organisms, telomeric DNA is composed of a tandem array of short sequences. For example, these repeats in *S. cerevisiae* consist of ∼350±75 bp of duplex TG_1–3_/C_1–3_A tracts. Internal to the TG_1–3_/C_1–3_A tracts are two classes of middle repetitive DNA elements, called X and Y' [1]. X elements with heterogeneous sizes ranged from 0.3∼3.75 kb exist in all chromosomes of *S. cerevisiae* [2], whereas one to four copies of Y'-long (6.7 kb) or Y'-short (5.2 kb) are found on about two-thirds of the telomeres. Telomeric DNA forms a heterochromatin structure with the Rap1p complex [3], [4]. Loss of telomeric protection can lead to the formation of end-to-end fusions, which directly disrupt the correct structure at telomere termini [5]. In addition to the inhibition of inappropriate repair responses, fusions or recombinations, telomeres need to be fully replicated by the action of telomerase to prevent end-replication problem [6], [7], [8], [9]. In *S. cerevisiae*, telomerase RNA and reverse transcriptase are encoded by *EST2* and *TLC1*, respectively. Although the majority of telomerase-minus *S. cerevisiae* cells enter cell cycle arrest eventually following the senescence stage [10], [11], survivors arise through *RAD52*-dependent homologous recombination (HR). Most survivors are type I, which have multiple tandem copies of the subtelomeric Y' element followed by very short terminal tracts of TG_1–3_/CA_1–3_ DNA [10], [12]. In a minor fraction of the survivors (type II), the lengths of telomere sequence are increased heterogeneously from several hundred base pairs to 10 kb or longer (Teng and Zakian, 1999). The generation of type I survivors depends on the presence of helicase Rad54, Rad51 that acts in strand invasion, Rad55-Rad57 complex that stabilizes recombination filament [13], and 5′-to-3′ exonuclease Exo1 [14]. The formation of type II survivors, on the other hand, requires MRX complex (Mre11, Rad50 and Xrs2), Rad59 for strand invasion, and the Sgs1-Top3 complex, which is involved in the process of HR [15], [16], [17], [18], [19].

Telomere attrition caused by lack of telomerase in *S. cerevisiae* has been shown to activate DNA damage checkpoints as they approach senescence, and this activation is also believed to be responsible for the premature growth arrest in these strains [20], [21]. The convergence of DNA damage and telomere checkpoints has also been revealed in mammalian cells [5], [22], [23], suggesting that DSBs and compromised telomere structure both impinge upon the ATM pathway that converges on a critical p53 nodal point. In the telomerase mutant mouse model, lack of p53 allows dysfunctional cells to avoid fates associated with checkpoint activation, including growth arrest and apoptosis [24], [25]. These results indicate that in mammals, as in yeast, telomere attrition resulting from lack of telomerase or alterations in telomere structure triggers characteristic DNA damage-initiated checkpoint. Additionally, not only do short telomeres share some of the biological and mechanistic features of DNA damage sites, they also exert effects on cellular response to DNA damage. Studies in telomerase-minus mice have suggested that telomere length correlates with organismal sensitivity to radiation [26], [27]. However it remains an open question of how the dysfunctional telomeres contribute to DSB sensitivity. Here we observed that yeast cells with short dysfunctional telomeres displayed *MEC1*-dependent hypersensitivity to DSBs and a robust redistribution of HR proteins from the endogenous DSBs to the telomeres, providing a molecular aspect of the relationship between aging and impaired DNA repairing capacity.

## Materials and Methods

See the Supplemental Data for additional experimental details.

### Strains and plasmids

All the yeast operations were performed by standard methods [28]. Yeast strains used in the study are listed in Table S1. All strains constructed in this study were isogenic to YPH501 (*MAT*a/*MATα ura3*-*52/ura3*-*52 lys2*-*801 amber/lys2*-*801 amber ade2*-*101 ochre/ade2*-*101 ochre trp1*Δ*63/trp1*Δ*63 his3*Δ*200/his3*Δ*200 leu2-*Δ*1/leu2-*Δ*1*), BY4743 (*MAT*a/α *his3*Δ*1/his3*Δ*1 leu2*Δ*0/leu2*Δ*0 LYS2/lys2*Δ*0 met15*Δ*0/MET15 ura3*Δ*0/ura3*Δ*0*), AGY673 [29], or BY4743 (Invitrogen). YPH499 *tlc1::LEU2* complemented with pRS316-*TLC1* was described previously [12]. For spotting assays, the *dnl4*::KanMX4 and *rad59*::*HIS3* mutants were constructed by transforming *dnl4*::KanMX4 and *rad59*::*HIS3* PCR fragments, respectively, into the YPH499 strain, and were crossed with YPH500 *tlc1*::*LEU2* to obtain heterozygous diploids. The *rad50* mutant and *mec1 sml1*and *tel1* mutants were described as previously [17], [30], [31] and were crossed with YPH500 *tlc1*::*LEU2* to obtain heterozygous diploids. The G418 resistant *rad9*, *rad24*, *chk1*, *pds1* and *dun1* mutants (Invitrogen) were crossed with the BY4741 *tlc1*::*HIS3* mutant (constructed by PCR-based replacement of the *TLC1* sequence) to obtain heterozygous diploids. The *pds1*::*LEU2* ts mutant was created in BY4742 by using pAY55 plasmid (a kind gift from Dr. D. Koshland) as previously described [32] and was crossed with *tlc1*::*HIS3* mutant to obtain heterozygous diploids. The *mrc1*::*HIS3* was constructed by PCR-based replacement of the *MRC1* ORF in BY4742 and the resulting strain was crossed with BY4741 *tlc1*::*LEU2 t*o obtain heterozygous diploids. All heterozygous diploids were sporulated for haploid single, double, or triple mutants.

For HOcs ChIP analysis, *tlc1* mutant was constructed by transforming AGY67 [29] with the *tlc1*:: *TRP1* PCR fragment amplified from STY161 (YPH499 *tlc1*::*TRP1*) genomic DNA using *TLC1*-5′ and *TLC1*-3′ as primer pairs. For telomere ChIP analysis, YPH499 UT strain containing modified chromosome VII-L was described previously [33], and both wild type and *tlc1*::*LEU2* spore colonies used in this study were derived from tetrad dissection of YPH501 UT *tlc1*::*LEU2*/*TLC1* diploids. For immunofluorescence assay, the Myc-tagged *RAP1* strain was constructed by double crossing over the chromosomal *RAP1* of YPH499 with a 13Myc PCR fragment from pFA6a-13Myc-KanMX6 [34]. This strain was transformed with pWJ1213 [35], a plasmid carrying Rad52-YFP and the *HIS3* marker (a kind gift from Dr. R. Rothstein) and its chromosomal *RAD52* was deleted by PCR-based replacement using a *RAD52*::*URA3* PCR fragment. The resulted strain was crossed with YPH500 *tlc1*::*LEU2* to obtain heterozygous diploids and sporulated for YPH499 wild type and *tlc1*::*LEU2* haploid cells containing KanMX6, *HIS3* and *URA3* markers.

All type I survivors were confirmed by the telomere restriction fragment (TRF) assay. All pre-senescent *tlc1* mutants used in this study were derived directly from spore colonies (about 25 generations). Genotype was confirmed by Southern blot, PCR and/or sequencing analysis. Primer sequences for PCR, real-time PCR amplification and sequencing are listed in Table S2.

### DNA damage sensitivity assays

For spotting assays, log-phase cultures were adjusted to an OD_600_ of 0.1 or 1 for type I survivors, and serial five-fold dilutions were plated on YPD followed by exposure to different temperatures, ionizing radiation (IR), UV light, or YPD containing various concentrations of glucose, bleomycin (BLM), methyl methanesulfonate (MMS), H_2_O_2_, or hydroxyurea (HU).

### Yeast culture condition, sample preparation, enzyme digestion, gel electrophoresis, and Southern blot analysis

Each spore colony was inoculated into YEPD and grown at 30°C. Several independent isolates for each genotype were analyzed. Spore cells were serially diluted into or restreaked onto YEPD medium. Liquid cultures were generated by inoculating spore colonies from the tetrad plate into 10 ml of liquid YEPD medium. Cultures were diluted repeatedly 1∶10,000 into fresh medium at 48 or 72 hours. A solid-plate study was performed by repeatedly streaking spore colonies from the tetrad plates on YEPD plates. Genomic DNA preparation and Southern blot analysis were performed as previously described [31], [36]. To examine the DNA from individual colonies, each colony was expanded in 2 ml of liquid medium to obtain DNA for Southern blot analysis. The DNA was digested with *Xho*I in order to observe the type I pattern or with a mixture of *Hae*III, *Hinf*I, *Hinp*II, and *Msp*I four-base cutters to observe the type II pattern. A 500-bp C_1–3_A fragment was randomly labeled with the Random Prime Labeling system (Invitrogen) and used in Southern hybridization. Data shown under “Results” are representative of two or more experiments from independent spore colonies.

### Protein extraction, trichloroacetic acid (TCA) precipitation and Western blot analysis

Yeast cells were grown overnight at 30°C in 5 ml YEPD medium and refreshed from OD_600_ = 0.2 to OD_600_ = 0.5 in 10 ml of YEPD. Cells were washed with 1 ml of sterile water and resuspended in 1 ml of cold 0.25 M NaOH/1% 2-mercaptoethanol, followed by incubation on ice for 10 min. Samples were added with 160 µl of 50% TCA and incubated on ice for additional 10 min. Cells were pelleted at full spin for 10 min and then washed with 1 ml of acetone. After air dry, 100 µl of 2x SDS sample buffer was added to resuspend protein extracts. Western blot analysis was performed as described [18] using an anti-Rad51 antibody (Santa Cruz sc-33626), an anti-Rad52 antibody (Santa Cruz sc-50445), or an anti-Pgk1 antibody (Molecular Probes A-6457) for detection.

### Chromatin immunoprecipitation (ChIP)

Yeast cells were grown overnight in 5 ml of YEPD at 30°C, washed twice with ddH_2_O, and refreshed to an OD_600_ = 0.2 in 100 ml YEP medium with 2% raffinose until mid-log phase (OD_600_∼0.5). One gram of galactose was then added into 50 ml of culture, while the other half was remained in YEP medium containing raffinose. After two hours of incubations at 30°C, cells were spin down and cross-linked with 1% formaldehyde and ChIP was conducted as described [37] using polyclonal anti-Rad51 and anti-Rad52 antibodies or normal rabbit IgG as a control. DNA was extracted and dissolved in 100 µl of TE. PCR primers specific to the *ASP3* gene (which is on a different chromosome from the *MAT*) were included as an internal control. ChIP samples were quantitated by real-time PCR, calculated as previously described [38], and were normalized to the value of each non-induced counterpart [29]. Data are expressed as “relative IP” using wild type as 100% enrichment. To collect samples for telomeric ChIPs, yeast cells were grown overnight in 5 ml of synthetic complete medium that lacked uracil at 30°C and refreshed to an OD_600_ = 0.2 in 100 ml of YEPD medium until mid-log phase (OD_600_∼0.5). Bleomycin was then added into 50 ml of culture to a final concentration of 7.5 mU/ml for 2 hr at 30°C, while the other half was remained in YEPD medium without treatment. Data for Rad proteins binding at telomeric regions were normalized to input DNA and expressed as the amount of VIIL (telomeric sequence on the left arm of chromosome VII) over that of *DDI2* gene (internal control). Samples were amplified in triplicate to obtain an average value and ChIPs were repeated at least three times for each strain.

### Immunofluorescence

All experiments were performed in 23°C to allow the YFP chromophore to form efficiently. Cells were fixed with 4% formaldehyde and processed for fluorescence microscopy as descried previously [39]. Rap1 foci were detected by using anti-Myc monoclonal antibody (9E10, Roche). To stain genomic DNA, 0.3 µg/ml DAPI was added to the mounting solution. All images were captured at 63-fold magnification using a Plan-Apochromat ×63, 1.4 NA objective lens. Cells were mounted and analyzed under a confocal microscope (Leica TCS SP5, Wetzlar). The illumination source was a 100-W mercury arc lamp. Images were acquired and pseudo-colored using Leica LAS AF software. For each field of cells, 22 images were obtained at 0.2-µm intervals along the z axis to allow inspection of all focal planes of each cell. At least 300 cells were counted to determine the percentage of cells with Rad52-YFP foci. For the percentage of Rad52-YFP foci colocalized with Rap1-Myc13, we calculated the percentage of YFP coinciding with anti-Myc signals, rather than the converse. Three persons independently scored at least 200 cells for 50% or more overlap of yellow and red traced areas, each of which was defined as a colocalization.

## Results

### Type I survivors are hypersensitive to DNA DSBs

**Figure 1:**
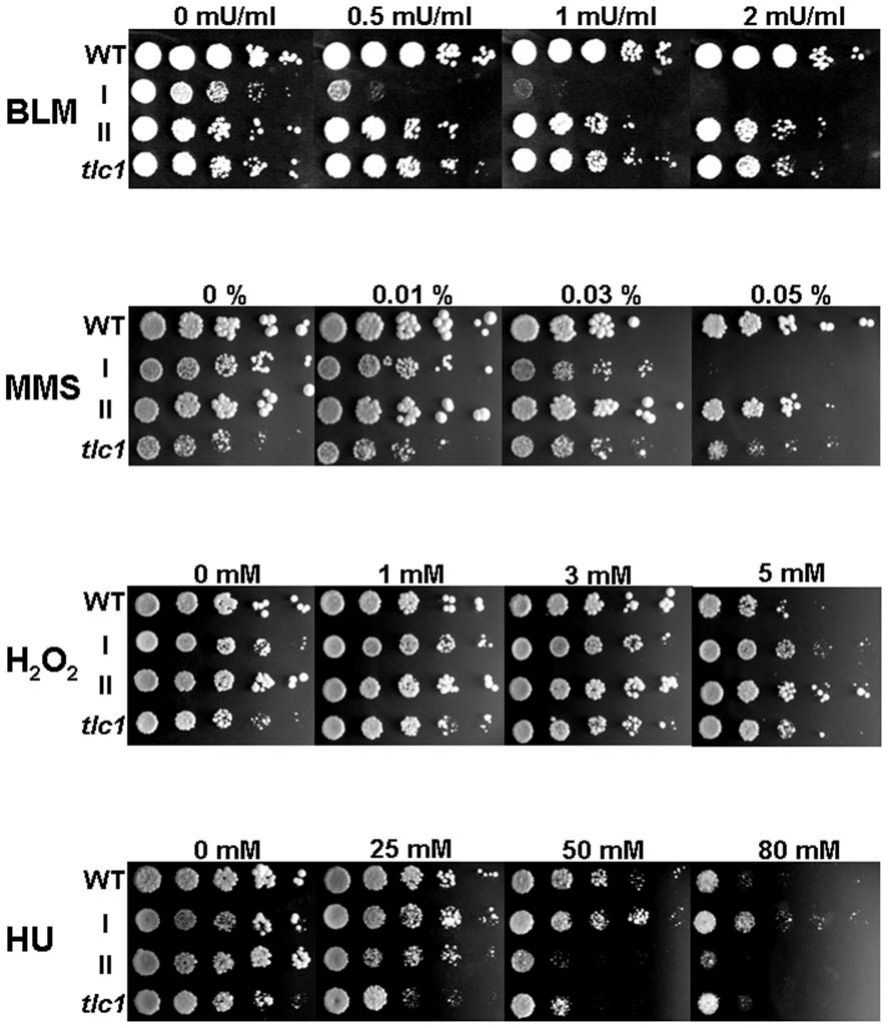
Type I survivors are hypersensitive to DNA-damaging agents. To measure the character of telomerase-minus cells, five-fold serial dilutions of isogenic wild type (WT), pre-senescent *tlc1* mutant, type I or type II strains were spotted onto YPD plates containing indicated drugs with denoted concentrations. The plates were incubated at 30°C for three days before photograph.

To characterize the property of telomerase-minus cells, we first challenged wild-type, pre-senescent telomerase-minus cells, senescent type I or type II survivors with different stresses. Interestingly, type I survivors were exquisitely hypersensitive to strand break-inducing agents, including BLM, MMS, IR, and UV light, but not to different temperatures, altered glucose concentrations, oxidative free radical-forming agent H_2_O_2_ or replication stress-inducing drug HU (Figure 1 and data not shown). The reason why type I cells were more resistant to HU than type II survivors is unclear. In order to confirm that growth inhibition induced by bleomycin was not restricted to a single source of type I survivors, we further tested the sensitivity of 448 type I survivors derived from nine independent *tlc1* spore colonies to bleomycin. After three days of incubation at 30°C, survival rates of these type I survivors on YEPD plates containing bleomycin (2 mU/ml) were scored (data not shown). 55% (248 of 448) of tested type I survivors were able to grow on plates containing bleomycin, while the rest 45% (200 of 448) were not. To determine if the Y'-short and Y'-long elements that form the major part of type I telomeres contribute to the generation of two distinct populations with different bleomycin sensitivity, genomic DNA of 24 sensitive and 24 resistant type I survivors was digested with *Xho*I and subjected to Southern blot analysis with a Y' probe. However, no apparent differences in Y' patterns between the two populations were observed (Figure S1), suggesting that Y'-long or Y'-short preference did not affect the bleomycin sensitivity of type I survivors.

### All type I survivors are eventually sensitive to bleomycin following sufficient passages

The results above drove us to quest whether the bleomycin sensitivity of type I cells was due to epigenetic events which rendered two interchangeable phenotypes, or constant events caused by irreversible genetic alterations. To distinguish these, we passaged both sensitive and resistant populations on solid plates for up to 12 streaks (240∼300 generations) and tested their sensitivity to bleomycin. At the 12^th^ streak, virtually all survivors (51/52) ceased to proliferate on plates containing bleomycin (Figure S2). This result implies that most of the type I survivors given sufficient outgrowth are eventually sensitive to bleomycin. Viewed in this finding, the sensitivity of type I can be regarded as a constant phenotype rather than a random event. It was notable that Southern blot analysis of Y' elements also showed no apparent differences in the distribution of the two types of Y' between the only resistant survivor and other sensitive ones at the 12^th^ streak (data not shown).

### Sensitive phenotype of type I survivors is attributed to the inactivation of telomerase

**Figure 2:**
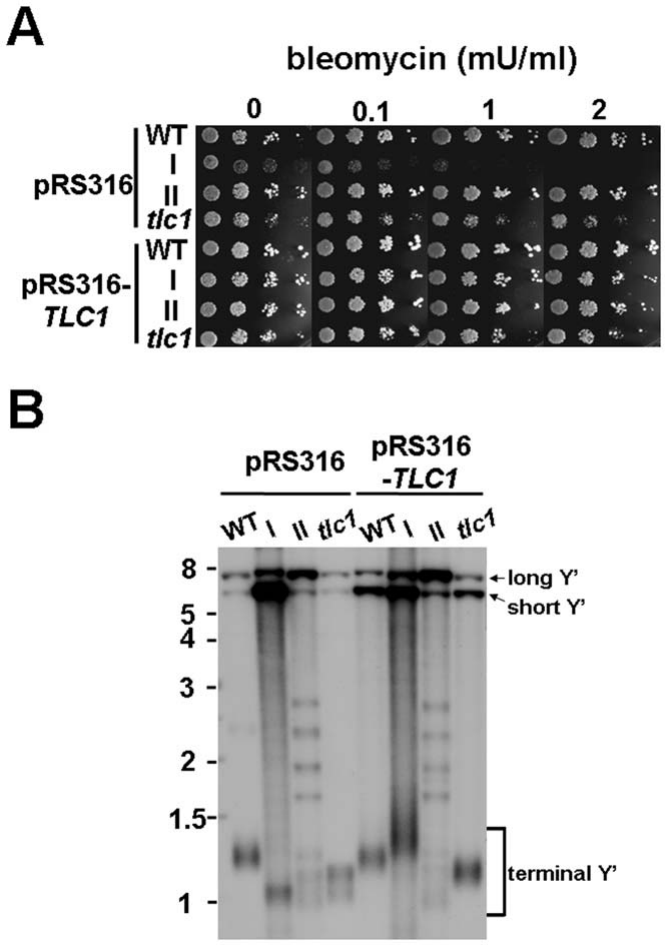
A CEN-based plasmid containing *TLC1* complemented the sensitive phenotype of type I survivors. (**A**) Wild-type, pre-senescent *tlc1* mutant, type I or type II strains carrying pRS316 or pRS316-*TLC1* plasmid were five-fold serially diluted and spotted onto YEPD plates containing bleomycin in various concentrations. (**B**) Southern blot analysis of the *Xho*I-digested genomic DNA isolated from strains described in panel A probed to detect telomeric sequences.

It has been reported previously that progressive telomere shortening leads to chromosome instability [40]. To test this possibility that critical genetic aberrations might arise from telomere attrition and thus contribute to the bleomycin-sensitive phenotype, the RNA component of telomerase from a CEN-based plasmid containing *TLC1* (pRS316-*TLC1*) was delivered into type I cells as well as control strains. As shown in Figure 2A, type I survivors became resistant to bleomycin after complemented with a plasmid-borne *TLC1*, suggesting that the sensitive phenotype of type I survivors was attributed to the inactivation of telomerase. A Southern blot analysis on the telomere pattern was also performed to monitor the change of telomere length in these strains (Figure 2B). Further measures on the rate of forward mutation to canavanine resistance (Can^r^) also showed no striking difference in the spontaneous mutation rate in type I survivors compared to control cells (Figure S3). These results indicate that rather than permanent genetic mutations, the loss of telomerase is more likely the major cause of sensitive response of type I survivors to DNA-damaging agents.

### Cells undergone progressive telomere attritions are susceptible to DSB attack

**Figure 3:**
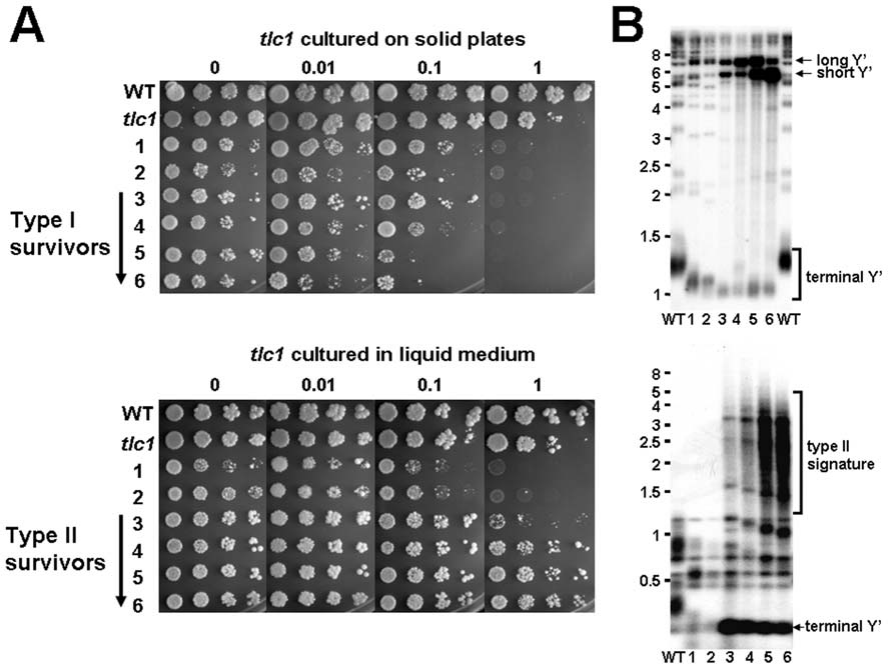
Dynamics of bleomycin response during *tlc1* mutant outgrowth. (**A**) Spot test analysis of serial 5-fold dilutions of the indicated strains on YEPD or YEPD containing indicated concentration of bleomycin. *tlc1* spore colonies were passaged either by restreaking on solid plates (top) or repeat dilutions in liquid medium (bottom). (**B**) Southern blot analysis of the *Xho*I- (top) or four-base cutter (bottom)-digested genomic DNA isolated from the strains in (**A**) probed with telomere sequences. The numbers in both panels indicate numbers of restreaking or dilutions. The arrows in (A) indicate the rise of type I (top) or type II (bottom) survivors.

In order to paint a detailed picture regarding exactly when the *tlc1* mutant becomes DSB-sensitive and what causes this distinct response between type I and type II survivors to DSBs, we tracked the change in the response to bleomycin from a *tlc1* spore colony till a survivor. Type I and type II survivors were obtained by repeatedly restreaking the *tlc1* spore colony on solid plates and continuous dilutions in liquid culture, respectively. Yeast colonies from each restreak (corresponds to 20–25 generations) or each dilution (corresponds to 13 generations) were analyzed by spot assays on YEPD plates with or without bleomycin (Figure 3A), and the timing of survivor formation was monitored by Southern blot analysis (Figure 3B).

When the *tlc1* mutant was maintained on solid plates, cells displayed sensitivity to bleomycin from the first streak, and they showed much lower viability on bleomycin plates for the following restreaks (Figure 3A, top), even after Y' amplification was generated at the 3^rd^ restreak, as it was evident by Southern blot analysis (Figure 3B, top). Unlike type I survivors, although the *tlc1* mutant also displayed sensitivity in the first two dilutions in liquid culture, increased resistance to bleomycin was observed immediately after the development of type II survivors (Figure 3A, lower), in which telomere lengthening occurred rapidly by recombination of TG_1–3_ tracts (Figure 3B, lower). Because the major difference between two subtypes of survivors is the application of distinct recombination pathways to reconstruct their chromosomal ends, we speculated that it is the shorter TG_1–3_ tracts in early senescent *tlc1* mutants and type I survivors that conferred their sensitivity to DSBs.

### Recruitments of Rad51 and Rad52 are reduced at an internal HO cut site but increased at telomeres in type I survivors

**Figure 4:**
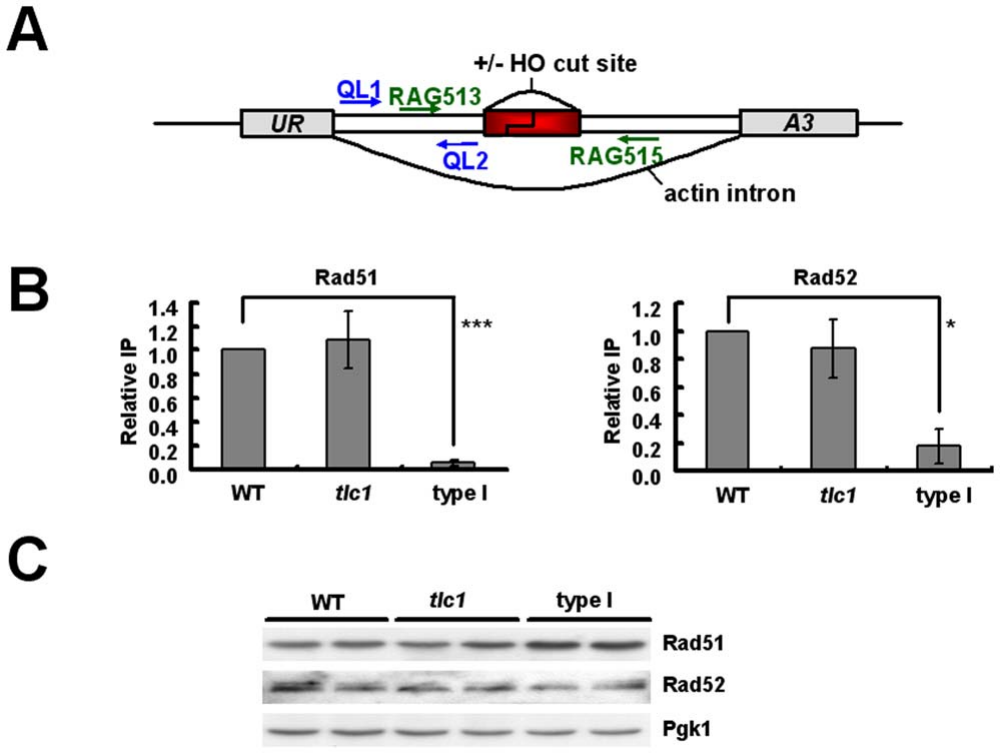
Recruitments of Rad51 and Rad52 at an HO cut site are reduced in type I survivors. (**A**) The structure of the *URA3* allele on *S. cerevisiae* chromosome V for these experiments. The position of various oligonucleotide primers (arrows) used for PCR and real-time PCR are shown. (**B**) ChIPs were performed and quantitated by real-time PCR as described in Materials and Methods. Error bars represent standard deviations for at least three independent experiments and are provided for these strains. Bindings of Rad51 and Rad52 to HOcs in type I cells differed from those in wild-type in a statistically significant manner (*P* values  = 0.0002 and 0.0115, respectively). These differences were not observed when a mock immunoprecipitation was performed with normal rabbit IgG (data not shown). (**C**) Rad51 and Rad52 expression levels in these strains were analyzed by Western blot analysis.

Previous works have shown that many DNA repair proteins are often associated with telomeres. We speculated that the titration of DNA repair proteins to shortened chromosomal ends might decrease repair capacity at bona fide DSB sites. Since DSBs as the major cytotoxic lesions caused by bleomycin are preferably repaired by HR [41], we subsequently examined whether Rad51 and Rad52 were less efficiently recruited to an induced DSB in type I survivors. We investigated the recruitment of Rad51 and Rad52 to a single DSB by ChIP. The experimental system is shown in Figure 4A: a functional *URA3* allele containing a copy of the actin intron as well as the Y–Z junction from the *MAT* **a** locus, including the recognition sequence and cleavage site for the HO endonuclease, was transformed into a yeast strain that has been deleted of all *MAT* related sequences and contains an integrated galactose-inducible HO endonuclease gene. Upon growth in galactose media, the HO endonuclease created a unique DSB in the middle of the *URA3* gene.

Wild type, *tlc*1 and type I strains were grown in YEP media with raffinose as the sole carbon source until mid-log phase. Galactose was then added to induce expression of the HO endonuclease. At various times after galactose addition, aliquots were removed and genomic DNA was isolated to analyze for the formation of a DSB at the *MAT* locus using a PCR assay. The induction kinetics showed that all strains remained only approximately 20∼27% of the original signal after two-hour induction (Figure S4). Figure 4B shows the quantitative results of ChIPs using a polyclonal antibody against Rad51 (left) or Rad52 (right). Immunoprecipitated DNA was analyzed by real-time PCR to detect *MAT* locus DNA from left side of the induced DSB (designated as “HOL”). PCR primers specific to the *ASP3* gene (which is on a different chromosome from *MAT*) were included as an internal control. Compared to wild type cells, type I survivors showed only 8% and 16% in bindings of Rad51 and Rad52, respectively, to the HO-induced DSB, while the pre-senescent *tlc1* mutant displayed similar results as wild type cells. To confirm that the reduced bindings of Rad51 and Rad52 in type I survivors was not due to lower protein expression levels, expression levels of both proteins were compared. As shown in Figure 4C, type I survivors expressed a higher level of Rad51 than that of wild type, and approximately the same level of Rad52 as that of wild type. This is consistent with the finding that Rad51 expression is up-regulated during senescence in genome-wide studies of expression response to telomere shortening [42], [43]. Therefore, it was not the lower protein expression that caused the decreased bindings of repair proteins to the HO cut site. To exclude the possibility that the reduced recruitments of HR proteins in type I survivors is caused by the different phases which WT and type I cells may be in, we conducted the ChIP experiment with both strains arrested in G2/M phase, and similar results were obtained (data not shown). From the data above, we propose that the sensitive phenotype to DNA-damaging agents in cells with short telomeres may be resulted from their impaired ability to recruit sufficient DNA repair proteins to damaged sites.

**Figure 5:**
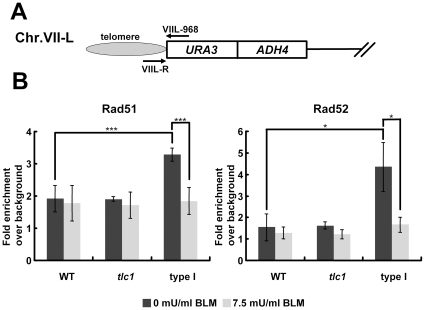
Sequestration of repair proteins at telomeres in type I survivors. (**A**) The left arm of chromosome VII-L in YPH499 UT strain was modified by integration of fragments containing the *URA3* and telomeric sequence at the *ADH4* locus. Telomeres are represented by hatched ovals; arrows indicate primers used for real-time PCR. (**B**) ChIPs were performed and quantitated by real-time PCR as described in Materials and Methods. Error bars represent standard deviations for at least three independent experiments. Before the addition of bleomycin (black bars), bindings of Rad51 and Rad52 to the VII-L telomere in type I cells differed from those in wild-type in a statistically significant manner (*P* values  = 5.36×10^−8^ and 0.010, respectively), and their bindings to the VII-L telomere was statistically indistinguishable in pre-senescent *tlc1* mutant and wild-type strains. In type I cells, bindings of Rad51 and Rad52 to the VII-L telomere before bleomycin treatment (black bars) differed from those after treatment (gray bars) in a statistically significant manner (*P* values  = 0.0004 and 0.014, respectively). This discrepancy was not observed in both pre-senescent *tlc1* mutant and wild-type strains.

To determine whether the gathering of repair proteins at short telomeres perturbed their recruitment to DSBs, the amount of Rad51 and Rad52 bound at the left telomere of chromosome VII (VII-L) was examined by ChIP in strains containing the modified chromosomes (Figure 5A). Since telomeres were constantly subject to recombinant events upon loss of telomerase and thus were likely to relocate or lose the sequence designed for quantification, the maintenance of the *URA3* marker in the telomere of type I survivors were confirmed by Southern blot before ChIP analysis (data not shown). ChIP samples were quantitated by real-time PCR. As shown in Figure 5B (left), there was a 3.2-fold enrichment of telomere sequences in the anit-Rad51 immunoprecipitate of type I cells, whereas both wild-type and pre-senescent *tlc1* mutant strains showed a 1.9-fold enrichment. Similar results were found with Rad52 (Figure 5B, right). While the short VII-L telomeric DNA was enriched 4.3-fold in the anti-Rad52 immunoprecipitate of type I survivors, there was only 1.5 and 1.6-fold enrichment in wild-type and pre-senescent *tlc1* mutant strains, respectively. As expected, the two-hour treatment with bleomycin (1.5 mU/ml) before ChIP samples were collected did not decrease the level of Rad52 bound to the telomere in type I survivors (data not shown). However, to exclude the possibility that the higher association of Rad proteins to the modified telomere in type I cells was attributed to some artifacts formed during the ChIP procedure, we further investigated whether these sequestered repair proteins can be released from telomeres to bona fide DSBs upon subjection to exogenous DNA damage. Towards this aim, before ChIP samples were collected, a portion of yeast culture was treated with high concentration of bleomcyin (7.5 mU/ml) for two hours. As a result, the bindings of both Rad proteins at VII-L reduced to the wild-type level in type I cells after treated with bleomycin (Figure 5B). In contrast, both wild-type and pre-senescent *tlc1* mutant strains displayed indistinguishable bindings of Rad proteins at VII-L before and after bleomycin treatment. All together, these results demonstrate that sequestration of Rad51 and Rad52 at short telomeres reduces the efficiency of their recruitments to an induced DSB site and that the mere onset of telomerase deprivation in pre-senescent *tlc1* mutant does not lead to the same level of biased distribution of Rad proteins as shown in type I survivors.

**Figure 6:**
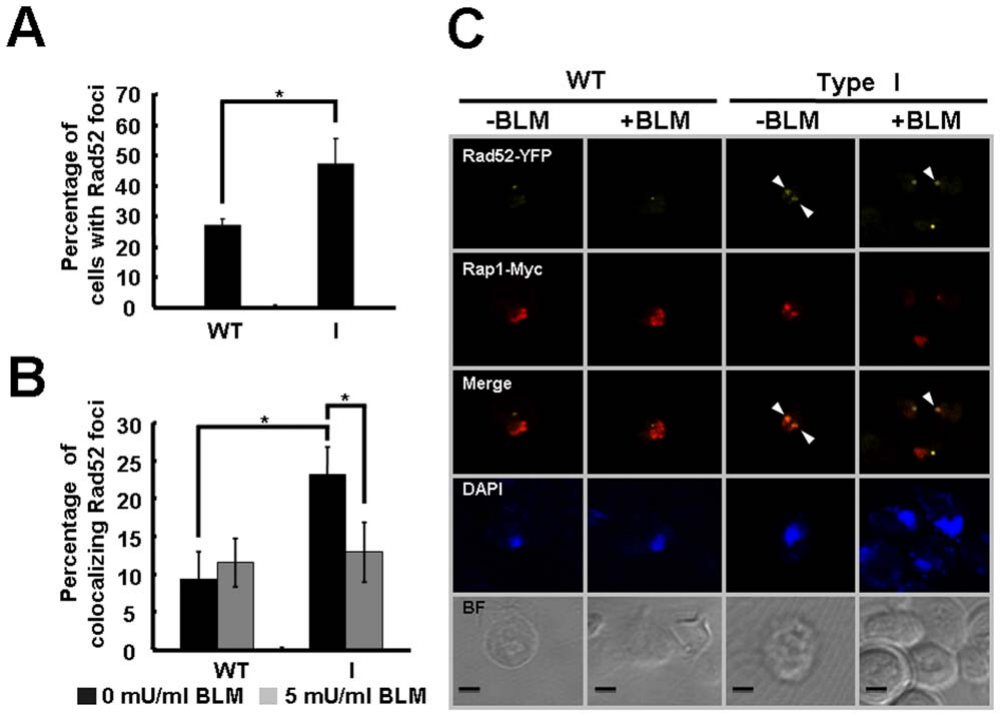
Localization of Rad52 to telomeres in cells with short telomeres. (**A**) The percentage of cells forming spontaneous Rad52-YFP foci was scored as described in Materials and Methods. (**B**) The percentage of Rad52-YFP foci colocalized with Rap1-Myc. Wild-type and type I cells expressing Rad52-YFP and Myc-tagged Rap1 were arrested in G1 with α-factor for 2.5 hours. Half of the culture was exposed to bleomycin for 30 min to generate DSBs. Cell treated with or without bleomycin were released into S-phase for 90 min. Before the addition of belomycin, the proportion of colocalized Rad52 in type I cells differed from that in wild-type in a statistically significant manner (*P* values  = 0.019), and in type I cells, the percentage of colocalization prior to bleomycin treatment (black bars) also differed from that after treatment (gray bars) in a statistically significant manner (*P* values  = 0.0232). Error bars represent standard deviations for at least three independent experiments. (**C**) Images of representative cells from the experiment in panel **B** show colocalization of Rad52 and Rap1 in type I cells. Scale bar, 2 µm. Arrowheads mark selected foci. BF, bright field.

To confirm that type I survivors sequester the key repair protein Rad52 to short telomeres at the cellular level, we investigated the redistribution of Rad52 in wild-type and type I cells by immunofluorescence using YFP-marked Rad52 and Myc-tagged Rap1. Since spontaneous Rad52 foci form mainly at chromosome breaks during normal DNA replication, to easily obtain signal, cells were arrested in G1 and then released into S-phase [44]. The percentage of cells presenting HR foci was determined microscopically. Consistent with the fact that short telomeres require the recombination protein Rad52 to be rescued, we found that type I survivors showed higher levels of Rad52 foci compared to WT cells (Figure 6A). To determine whether the higher level of spontaneous Rad52 foci formed in type I cells reflects HR proteins being sequestered at short telomeres, colocalization of Rad52 with the telomeric DNA-binding protein Rap1 (tagged with Myc) was investigated. While wild-type strains formed only 9.3% of the Rad52 foci colocalized with a Rap1 focus, type I cells displayed more than 20% colocalization prior to bleomycin treatment (Figure 6B, black bars) and this level of colocalization slightly decreased when subjected to DSBs (Figure 6B, gray bars). These results confirm our ChIP data (Figure 5) that HR proteins were “hyper-recruited” to the telomeres in Type I survivors and that high concentration of bleomycin released HR proteins from their associated telomeres, even though this redistribution may not be sufficient to repair DNA damage caused by excessive dose of bleomycin.

### The sensitivity of type I cells to bleomycin behaves in a checkpoint-dependent manner

Previous studies have indicated that the short telomeres cause cell cycle arrest [20], [21], [45], [46], [47], suggesting that compromised telomeres are recognized as damaged DNAs and are capable of triggering the DNA repair machinery during an arrest. Several proteins in the HR pathways and factors involved in the DNA damage machinery have been reported to associate with the ALT pathway or to participate in the process when compromised telomeres are detected as DSBs [31], [46], [48]. Therefore, we are interested in whether these genes impinge on the bleomycin sensitivity of type I survivors.

**Figure 7:**
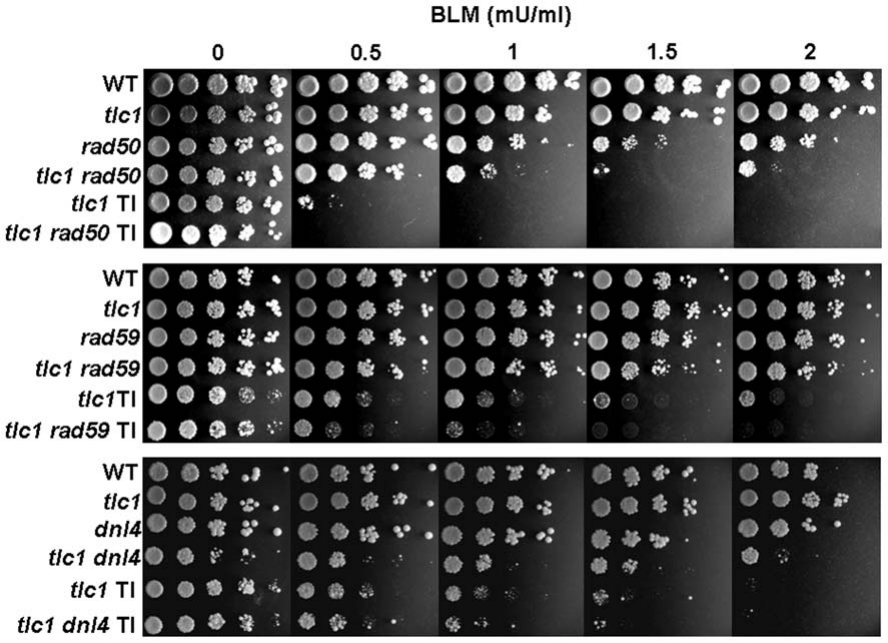
Deletion of *RAD50* or *RAD59* slightly altered the sensitivity of type I survivors. Exponentially growing cells were five-fold serially diluted, spotted onto YPD plates containing bleomycin with indicated concentrations and incubated at 30°C for 3 days. TI represents type I survivors derived from each mutant spore colonies, and all the others are pre-senescent spore colonies.

We first asked whether factors controlling the fate of repair in telomerase-minus cells contribute to this sensitivity. Both *RAD50* and *RAD59*, which belong to the *RAD52* epistasis group, are required for telomere maintenance in telomerase-minus cells and are not essential for type I pathway [17], [19]. Elimination of *RAD50* or *RAD59* in type I cells was subjected to spotting assays as described in Figure 1. As shown in Figure 7, deletion of either *RAD50* or *RAD59* slightly increased the sensitivity of type I cells to bleomycin, whereas abrogation of a major nonhomologous end joining (NHEJ) component *DNL4* resulted in no further phenotypic alterations. These data further underline the role of HR proteins in the sensitivity to DNA damage of type I cells.

**Figure 8:**
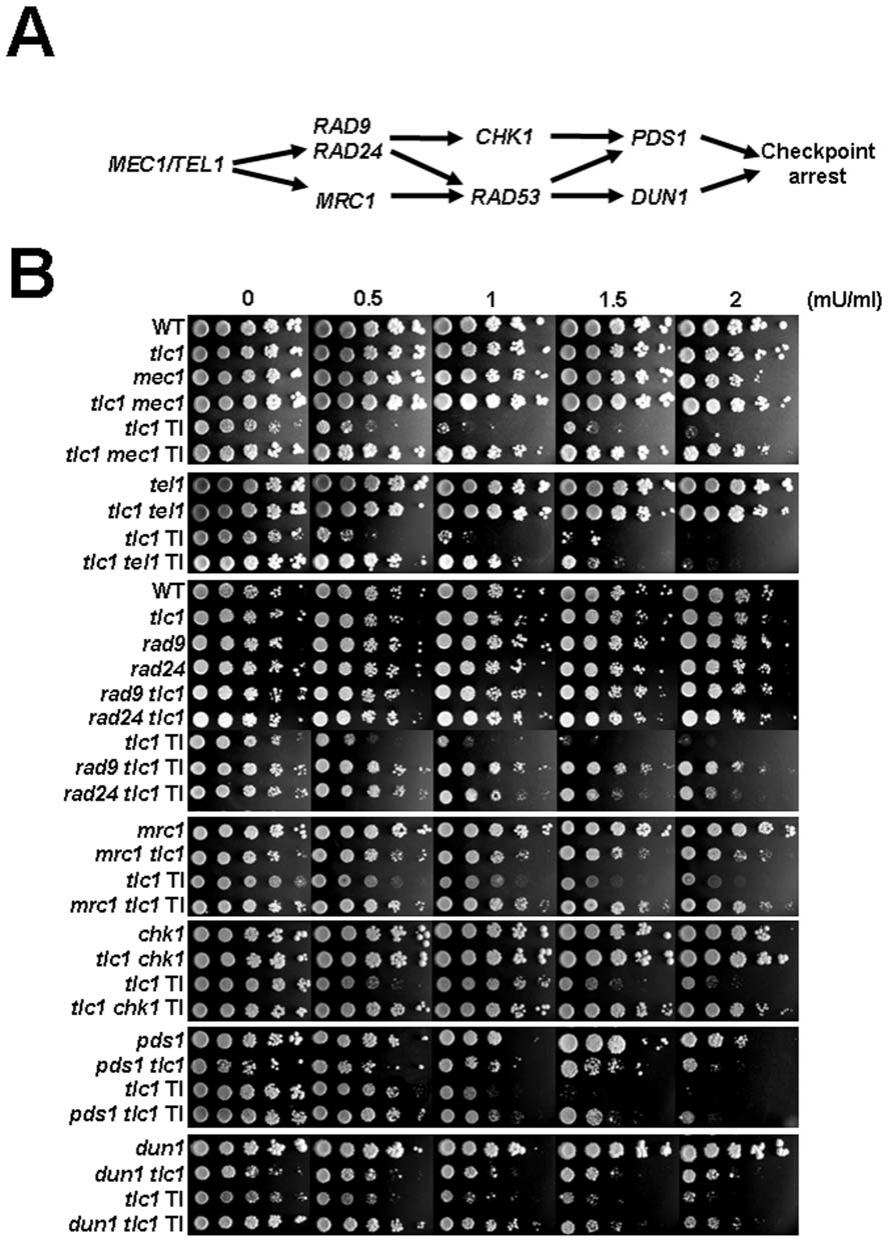
Type I sensitivity to bleomycin is in a largely checkpoint-dependent manner. (**A**) Signaling of *MEC1*/*TEL1*-regulated checkpoint pathway [56]. (**B**) Exponentially growing cells were five-fold serially diluted and spotted onto YPD plates containing bleomycin with indicated concentrations. Except for the plates shown in the last panel, which were incubated at 25°C, all of the strains were incubated at 30°C for three days. TI represents type I survivors derived from each mutant spore, and all the others are pre-senescent spore colonies.

Given that we have additionally observed a chronic cell cycle delay and persistent Rad53 phosphorylation in type I survivors (Figure S5), spotting assays were performed to investigate if checkpoint activation (Figure 8A) correlates with type I sensitivity to bleomycin. Strikingly, ablation of *MEC1* greatly enhanced type I survival rate in response to bleomycin, whereas deletion of *TEL1* only mildly promoted its survival (Figure 8B). To ensure that these results were accompanied with modified checkpoint competence, the phosphorylated level of Rad53 in various strains was determined by Western blot analysis. Indeed, the absence of *MEC1* completely abolished persistent checkpoint activation in type I survivors (Figure S5). Moreover, *MEC1* deletion largely hampered Rad53 phosphorylation induced by bleomycin treatment in type I survivors (Figure S5). Since similar results were not observed in *tel1* deletion mutants, the persistent checkpoint activation, the associated cell cycle arrest and the bleomycin sensitivity might be mainly dependent on *MEC1*. These observations prompted us to further study if the ablation of *MEC1* downstream genes (Figure 8A) surmounts the sensitivity phenotype of type I cells. As shown in Figure 8B, deletion of *RAD9*, *RAD24*, *MRC1*, *CHK1*, *DUN1* or *PDS1* (partially deleted as a temperature sensitive mutant, see Materials and Methods) promoted the proliferation of type I cells on plates containing bleomycin. Notably, we were unable to determine whether *RAD53*-deleted cells display the same effect since the spore colonies were too sick to survive on YEPD plates during the second restreak and no type I survivors could be assayed. Altogether, these results indicate that a Mec1-related checkpoint signal pathway regulates the hypersensitivity to DSBs in cells with short telomeres.

## Discussion

The impact of telomere shortening on senescent budding yeast upon loss of telomerase function is rather difficult to estimate because these cells display decreasing vitality and eventually die within a narrow timeframe. In this study, we observed that type I survivors are exclusively hypersensitive to DSBs (Figure 1). We have proved that this phenotype was not caused by some permanent genetic distortions since it could be complemented by a plasmid-borne *TLC1* and elevated mutation rates were not detected in type I cells (Figure 2 and S3). We further discovered that the sensitive response to DSBs is not only restricted to type I survivors, but also applies to *tlc1* senescent cells before any survivor formation (Figure 3A). Once TG_1–3_ tracts are lengthened through the recombination pathway, such as in type II survivors, cells can be recovered from the DSB-sensitive phenotype (Figure 3A). Southern blot analysis (Figure 3B) demonstrated that the overall TG_1–3_ tracts of senescent *tlc1* mutants and type I survivors are relatively shorter than those of wild type or type II survivor strains. This indicates a correlation between short telomeres and ineffective DSB repair.

Our finding of the reduced binding of HR proteins to a single cut site along with the observation of increased association of HR proteins with telomeres in type I survivors (Figures 4, 5, and 6) provides a potential mechanistic basis for the impaired DSB response in organisms with dysfunctional telomeres. Similar observations have been proposed in two recent studies [49], [50], both of which reported a robust Rad52 localization with the telomere in the *est2* mutant. Meyer and Bailis [49] have also demonstrated a correlation between this sequestration of Rad52 to telomeres in the *est2* mutant and reduced localization to an induced DSB formation. One apparent difference with our results is that the pre-senescent *est2* mutant strain in the Meyer *et al.* experiments showed increased binding of Rad52 to the telomere, whereas we did not observe this in our pre-senescent *tlc1*mutant. The discrepancy can be explained by the divergent strain backgrounds that generate different rates of senescence. Additionally, our ChIP results are consistent with the observation that pre-senescent *tlc1* mutant is not as sensitive as senescent cells or type I survivors to bleomycin (Figure 3A).

The idea of competition between DNA damage sites has also been reported elsewhere: the inhibitory effect of NHEJ on the initiation of DSB resection in G1 phase is able to be suppressed by four or more DSBs, indicating that some components of NHEJ may become limiting in the presence of multiple breaks [51]. Because shortened telomeres mimic DSBs, we speculate that DSB repair proteins, either for HR or NHEJ, might become limiting as well in the presence of shortened telomeres that resemble DSB sites.

One surprising finding was that the *MEC1* removal leads to a dramatic increase in the bleomycin resistance in type I survivors (Figures 8B), indicating that type I sensitivity to bleomycin is dependent on *MEC1*. Type I survivors persistently displayed phosphorylated Rad53 even in the absence of bleomycin and the bleomycin-induced Rad53 mobility shift was almost abolished in conjunction with the abrogation of *MEC1* gene (Figure S5), suggesting that type I sensitivity to DSBs might be attributed to the persistent Mec1-related checkpoint activation. These findings were quite striking, because checkpoint activation is normally expected to coincide with improved damage resistance. However, here we observed that diminished checkpoint activation by deleting *MEC1* dramatically promoted bleomycin tolerance of type I survivors. Nonetheless, similar results have been reported previously [52], as the abolished Rad53 phosphorylation in *mec1* mutants is suppressed by deletion of *MDT1*. This improved checkpoint activation paradoxically worsened bleomycin tolerance compared to *mec1* single mutants. Moreover, Alabert et al. have reported similar conflicting observations that Mrc1-dependent checkpoint activation prevents homolgous recombination at DNA DSBs [44]. They proposed that ssDNA exposed at stalled forks is the key signal to recruit HR machinery and suppress potentially dangerous HR at DSBs, since ssDNA is already present at the stalled fork and DSB repair depends on the formation of the 3′ ssDNA overhangs. These findings prompt us to hypothesize that short telomeres distinguish themselves from DSBs through exposed 3′ overhangs to trigger Mec1-dependent checkpoint activation and thus prevent HR at DSBs that still need resection. In fact, the idea that Mec1 is required to recognize signals emanating from short telomeres was supported by a recent ChIP result showing that Mec1 interacts physically with critically short telomeres, thereby sensing and transducing the signal for G2/M cell-cycle arrest [53]. In this study, even though type I survivor formation seems to be one major alternative pathway to repair the damage caused by dysfunctional telomeres, these survivors, nevertheless, resemble the senescent cells that suffer from a prolonged arrest. This prolonged arrest indicates an attempt to repair “chromosomal end damage” in type I cells. Combined with the ChIP and IF data, it is tempting to speculate that in the absence of Mec1, short chromosomal ends are not properly recognized as DNA damage sites, thereby becoming less competitive for DNA repair proteins that are normally recruited to bona-fide DSBs (Figure S6). Further investigations are needed to clarify this possibility. Altogether, this study suggests that the translocation of Rad51, Rad52, and likely other repair factors to telomeres contributes to the Mec1-dependent hypersensitivity to DSBs in telomerase deficient cells.

Studies of the role of telomerase in the maintenance of genome integrity in mammalian cells have provided insight into the molecular aspects in aging and cancer, and in particular, epithelial carcinogenesis [54]. A growing number of evidence also supports that various pathways of DNA repair become less efficient in aged cells [55]. The work presented here suggests another mechanism by which aged organisms may suffer from increased rates of unrepaired DNA damage and its concomitant risk of cancer. Careful considerations of the ways in which effective therapeutics are designed may help for the treatment and prevention in the aged.

## Acknowledgments

We thank Dr. Tsai-Kun Li for his discussion and critical comments. We also thank Drs. A. Gabriel, V. Zakian, D. Koshland, J. Diffley, A. Sugino, and R. Rothstein for their plasmids, strains and antibodies. Finally, we would like to thank our anonymous reviewers that helped clarify several vital points in the manuscript.

## Supporting Information

Figure S1 Patterns of Y' telomeres are indistinguishable between sensitive and resistant type I survivors. Equal amounts of the *Xho*I-digested genomic DNA isolated from wild-type and type I survivors of *tlc1* mutants (6^th^ streak) were loaded for Southern blot analysis, using a Y' fragment as a probe [1]. BLM^R^ indicates strains that are resistant to bleomycin at 2 mU/ml and BLM^S^ indicates sensitive strains. Arrows denote the positions of Y'-long and Y'-short elements. Marker sizes are indicated at the left (Kb).
(4.25 MB TIF)

Figure S2 Survival rates of early type I survivors on bleomycin plates. Survival rates were measured as the number of viable patches on YEPD containing 2 mU/ml of bleomycin over that on YEPD without bleomycin. Type I survivors from nine independent spore colonies were examined. A total of 447 survivors were scored for the 6^th ^streak, 52 for the 9^th ^ and 52 for the 12^th ^ streaks, respectively.
(1.06 MB TIF)

Figure S3 Measurement of mutation rates. (A) Assay for mutation rate measurement. Strains harbor a *can1* mutation are resistant to canavanine. (B) To measure the rate of forward mutation to canavanine resistance (Can^r^), at least six yeast cultures were started from single colonies and grown to stationary phase in 10 ml liquid YEPD medium. Cells were plated with appropriate dilutions onto complete medium containing L-canavanine (60 mg/ml) and lacking arginine for Can^r^ mutant count, and onto complete medium lacking arginine for viable count. The *pol2-4* (STY1609) mutant, previously reported to express a mutator phenotype [2], was used as a positive control. YIpBI (kindly provided by Dr. A. Sugino) was used to create the *pol2-4* mutant in YPH499 background by the method previously described [3]. Mutation rates were determined by the method of the median [4].
(0.98 MB TIF)

Figure S4 Kinetics of DSB induction at the HO cut site. Galactose (2%, w/v) was added to cells in mid-log phase in order to induce HO endonuclease expression. Genomic DNA was purified at various time points, and PCR was performed using primers RAG513 and RAG 515 that flank the HO cut site (HOcs) from 114 bp CEN distal of the HOcs to 946 bp CEN proximal. The DSB was detected as a loss of PCR product. Primers specific to the *ASP3* gene were included in the PCR as a control. DSB bands were quantitated by the ImageQuant software, normalized to the *ASP3* bands, and indicated as the percentage of starting signal (“% remaining product”) at the bottom of the panel.
(2.53 MB TIF)

Figure S5 Rad53 phosphorylation and the sensitivity to bleomycin of type I survivors is mainly dependent on Mec1. Rad53 phosphorylation was assayed by Western blot analysis. Proteins were prepared from strains in Figure 8B (top two panels) using trichloroacetic acid precipitation as described in Materials and Methods. Samples were separated on 7% SDS-polyacrylamide gels and transferred to nitrocellulose membranes. The membranes were incubated with a 1∶1000 dilution of anti-Rad53 antibody (gift of J. Diffley), followed by incubation with the secondary antibody. Membranes were developed using ECL chemiluminescence (GE) and exposed to autoradiographic film. It should be noted that Rad53 was partially phosphorylated in type I survivors before bleomycin treatment (5 mU/ml, 3 hours) and that was suppressed by *MEC1* deletion.
(2.79 MB TIF)

Figure S6 The distribution of Rad proteins in cells with short telomeres. Rad51 (red triangles) and Rad52 (blue ovals) are sequestered at chromosome ends in cells with short telomeres.
(2.50 MB TIF)

Table S1 Strains used in this study
(0.09 MB DOC)

Table S2 Oligonucleotide primers
(0.04 MB DOC)

Supporting Information References S1 (0.03 MB DOC)
