# Supplementary figures and images for: Recruitment of Rad51 and Rad52 to Short Telomeres Triggers a Mec1-Mediated Hypersensitivity to Double-Stranded DNA Breaks in Senescent Budding Yeast
Source: PLoS One. 2009 Dec 14;4(12):e8224. doi: 10.1371/journal.pone.0008224 (PMC2790616; doi:10.1371/journal.pone.0008224)

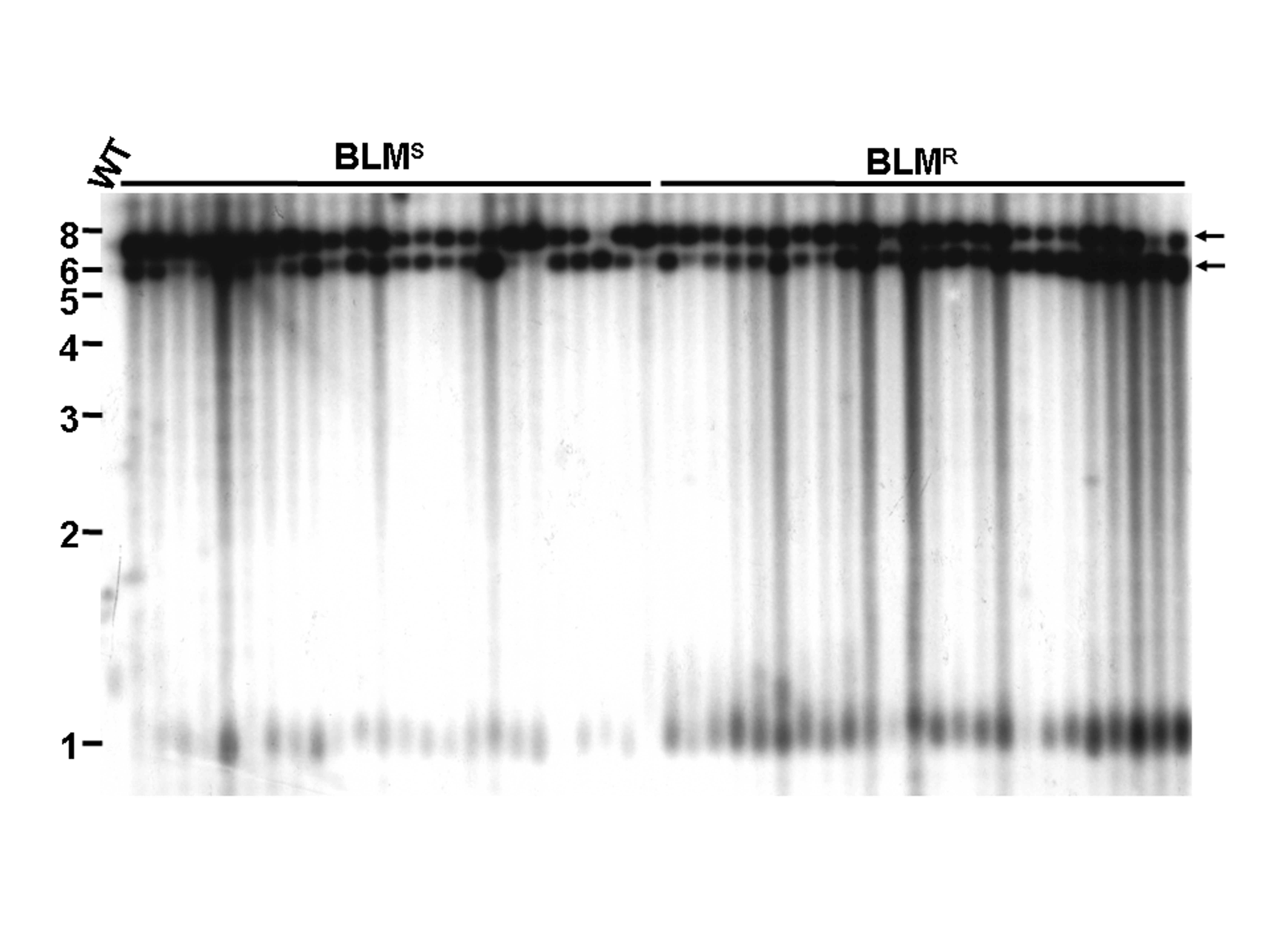

Supplement: Figure S1 — Patterns of Y' telomeres are indistinguishable between sensitive and resistant type I survivors. Equal amounts of the XhoI-digested genomic DNA isolated from wild-type and type I survivors of tlc1 mutants (6th streak) were loaded for Southern blot analysis, using a Y' fragment as a probe [1]. BLMR indicates strains that are resistant to bleomycin at 2 mU/ml and BLMS indicates sensitive strains. Arrows denote the positions of Y'-long and Y'-short elements. Marker sizes are indicated at the left (Kb). (4.25 MB TIF) [file pone.0008224.s001.tif]

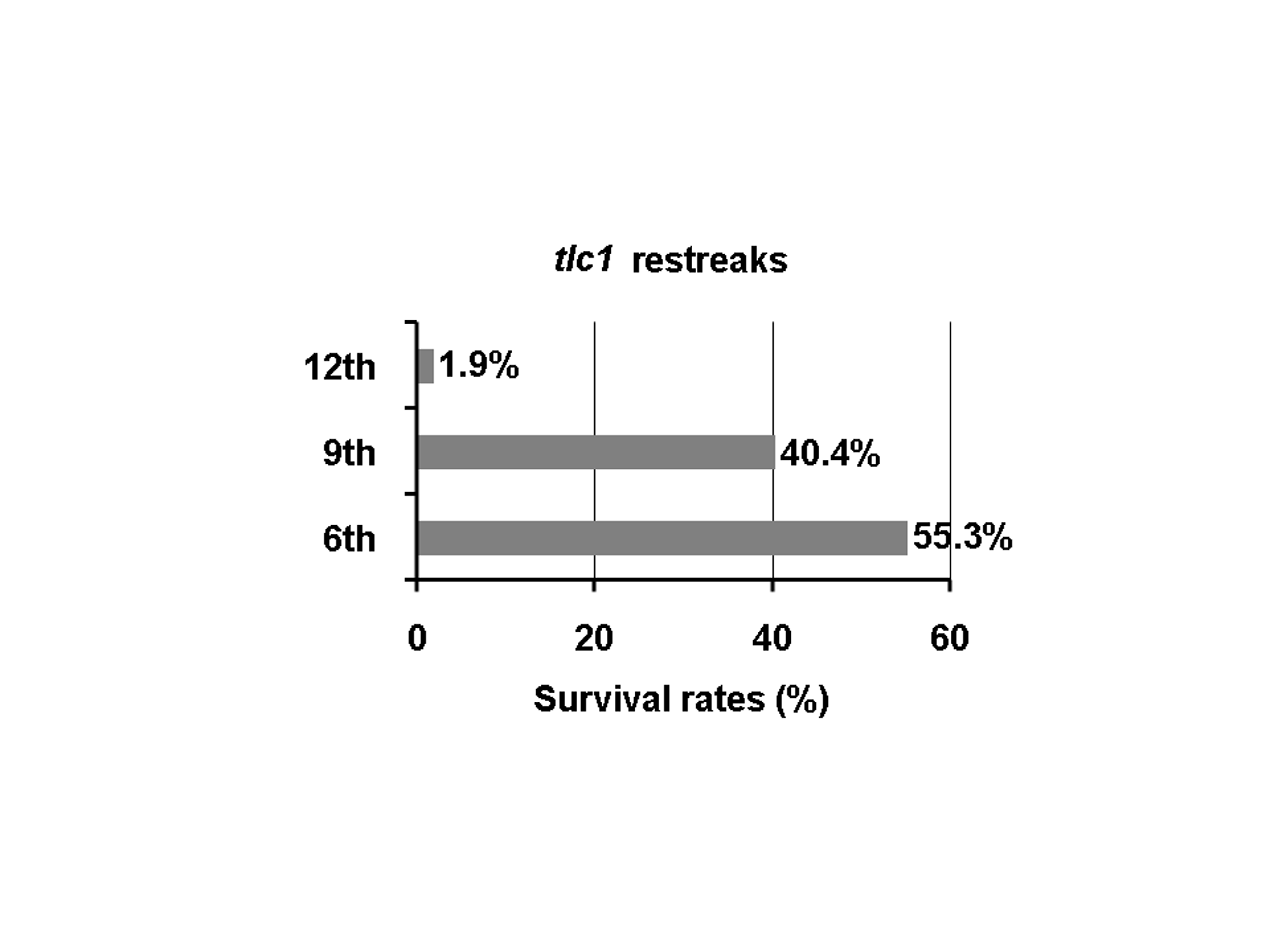

Supplement: Figure S2 — Survival rates of early type I survivors on bleomycin plates. Survival rates were measured as the number of viable patches on YEPD containing 2 mU/ml of bleomycin over that on YEPD without bleomycin. Type I survivors from nine independent spore colonies were examined. A total of 447 survivors were scored for the 6th streak, 52 for the 9th and 52 for the 12th streaks, respectively. (1.06 MB TIF) [file pone.0008224.s002.tif]

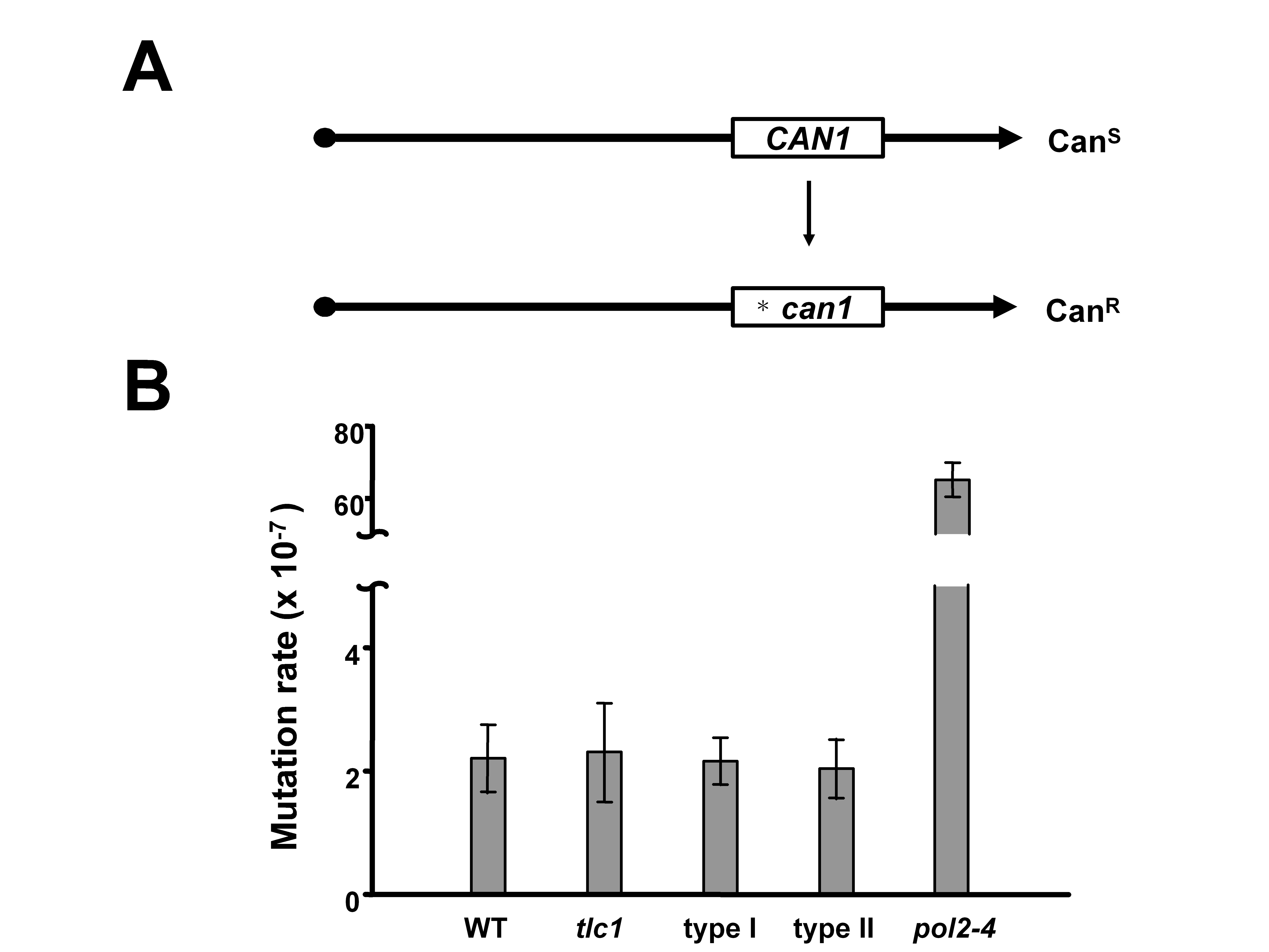

Supplement: Figure S3 — Measurement of mutation rates. (A) Assay for mutation rate measurement. Strains harbor a can1 mutation are resistant to canavanine. (B) To measure the rate of forward mutation to canavanine resistance (Canr), at least six yeast cultures were started from single colonies and grown to stationary phase in 10 ml liquid YEPD medium. Cells were plated with appropriate dilutions onto complete medium containing L-canavanine (60 mg/ml) and lacking arginine for Canr mutant count, and onto complete medium lacking arginine for viable count. The pol2-4 (STY1609) mutant, previously reported to express a mutator phenotype [2], was used as a positive control. YIpBI (kindly provided by Dr. A. Sugino) was used to create the pol2-4 mutant in YPH499 background by the method previously described [3]. Mutation rates were determined by the method of the median [4]. (0.98 MB TIF) [file pone.0008224.s003.tif]

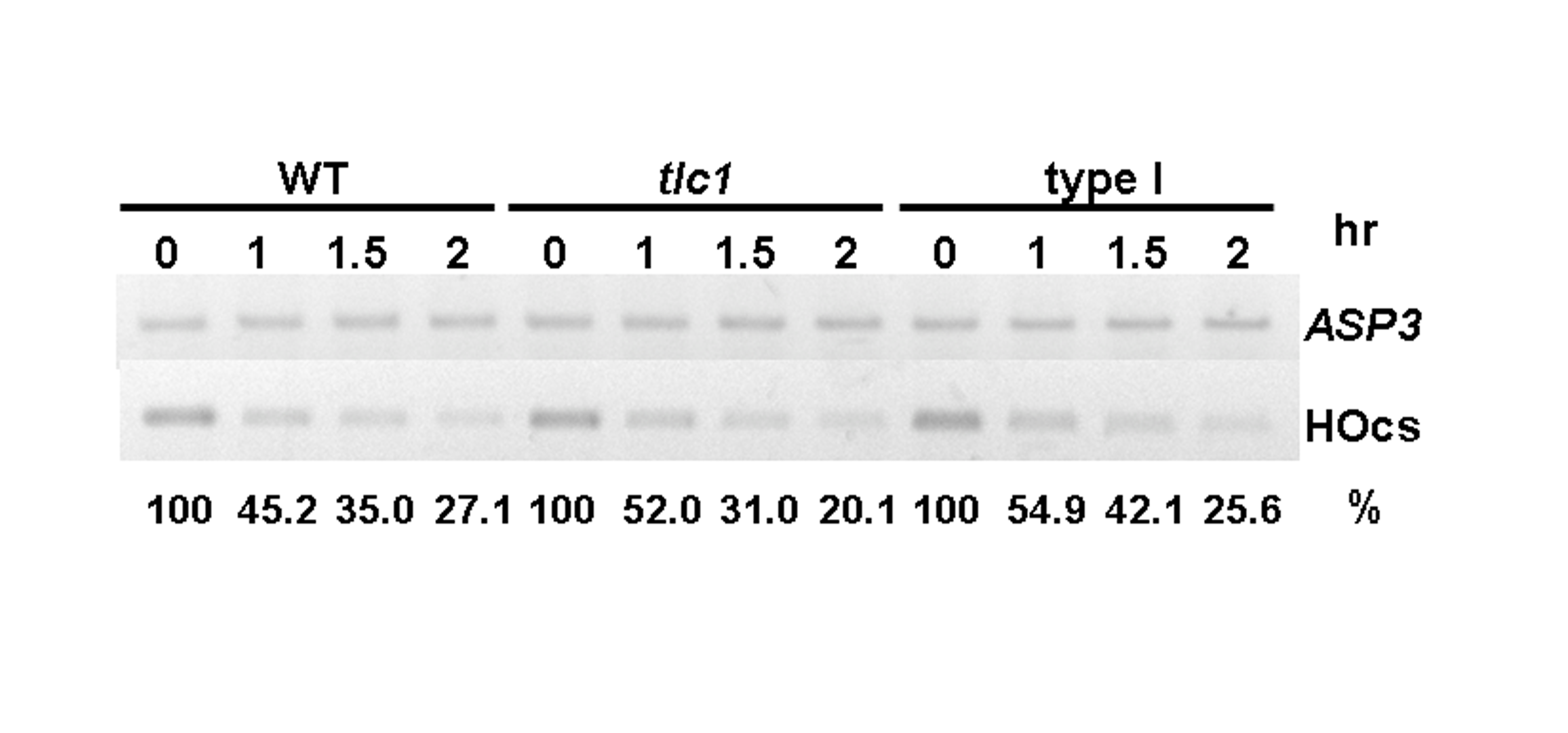

Supplement: Figure S4 — Kinetics of DSB induction at the HO cut site. Galactose (2%, w/v) was added to cells in mid-log phase in order to induce HO endonuclease expression. Genomic DNA was purified at various time points, and PCR was performed using primers RAG513 and RAG 515 that flank the HO cut site (HOcs) from 114 bp CEN distal of the HOcs to 946 bp CEN proximal. The DSB was detected as a loss of PCR product. Primers specific to the ASP3 gene were included in the PCR as a control. DSB bands were quantitated by the ImageQuant software, normalized to the ASP3 bands, and indicated as the percentage of starting signal (“% remaining product”) at the bottom of the panel. (2.53 MB TIF) [file pone.0008224.s004.tif]

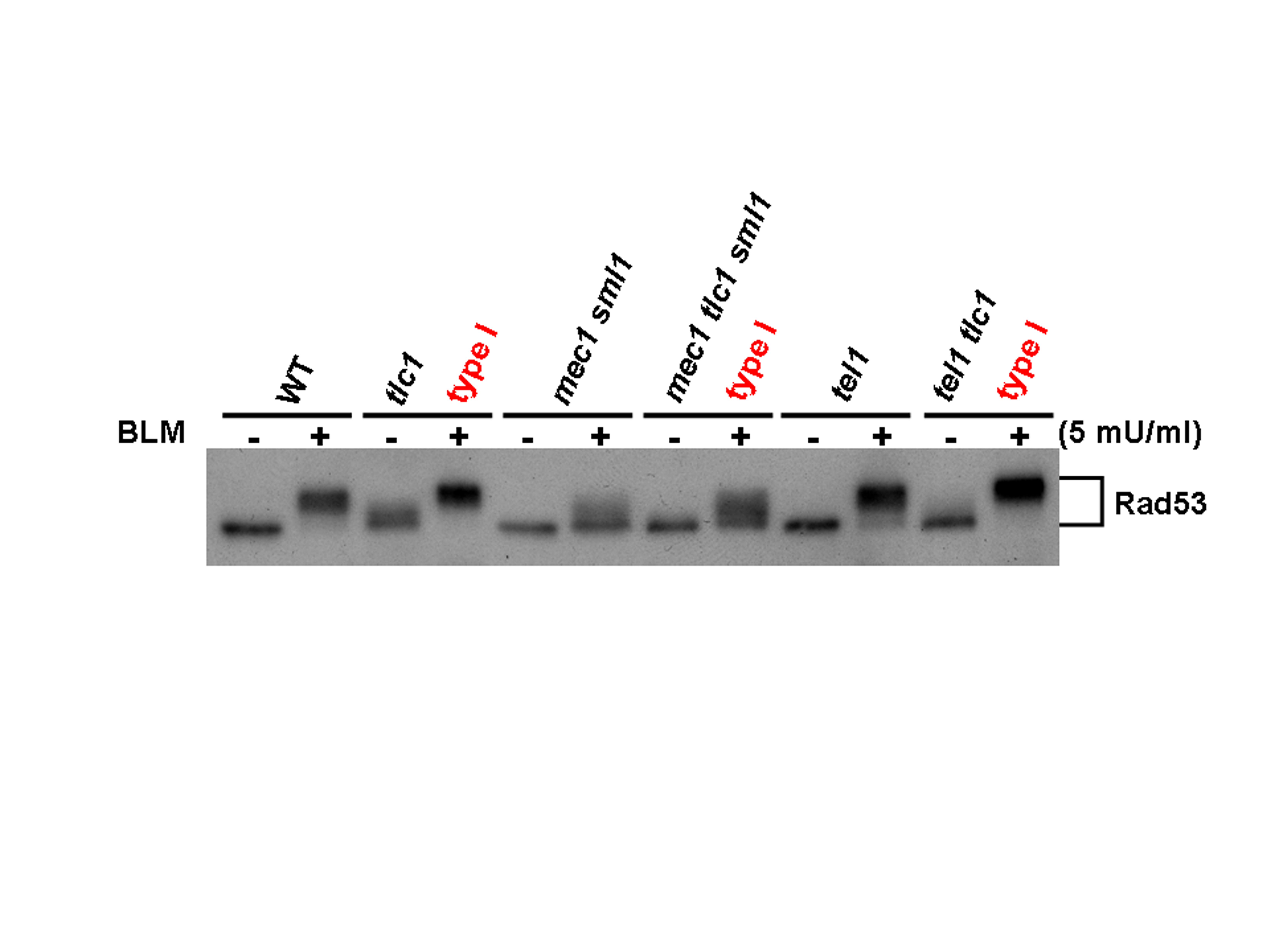

Supplement: Figure S5 — Rad53 phosphorylation and the sensitivity to bleomycin of type I survivors is mainly dependent on Mec1. Rad53 phosphorylation was assayed by Western blot analysis. Proteins were prepared from strains in Figure 8B (top two panels) using trichloroacetic acid precipitation as described in Materials and Methods. Samples were separated on 7% SDS-polyacrylamide gels and transferred to nitrocellulose membranes. The membranes were incubated with a 1∶1000 dilution of anti-Rad53 antibody (gift of J. Diffley), followed by incubation with the secondary antibody. Membranes were developed using ECL chemiluminescence (GE) and exposed to autoradiographic film. It should be noted that Rad53 was partially phosphorylated in type I survivors before bleomycin treatment (5 mU/ml, 3 hours) and that was suppressed by MEC1 deletion. (2.79 MB TIF) [file pone.0008224.s005.tif]

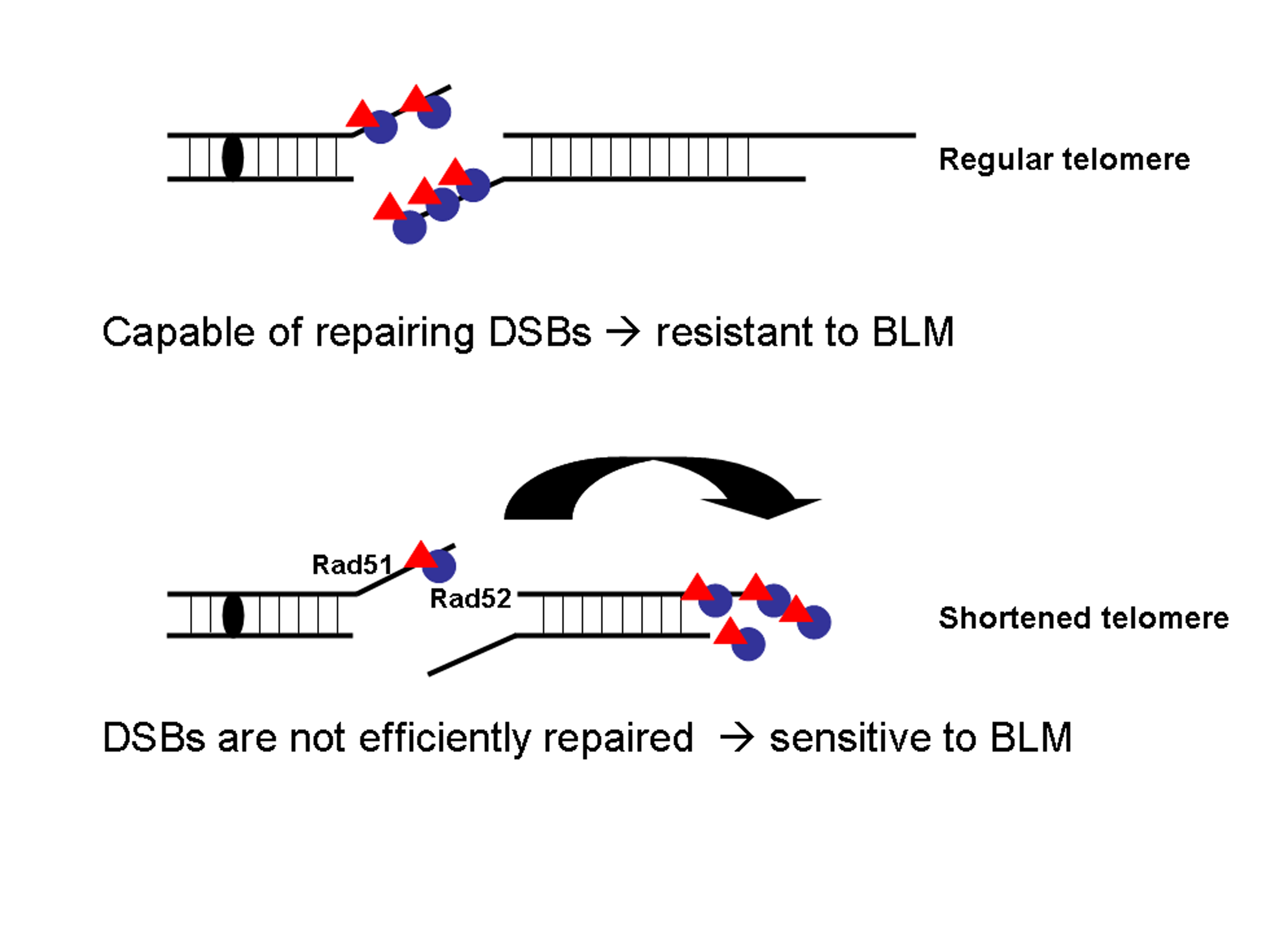

Supplement: Figure S6 — The distribution of Rad proteins in cells with short telomeres. Rad51 (red triangles) and Rad52 (blue ovals) are sequestered at chromosome ends in cells with short telomeres. (2.50 MB TIF) [file pone.0008224.s006.tif]
