# Supplementary material for: Recruitment of Rad51 and Rad52 to Short Telomeres Triggers a Mec1-Mediated Hypersensitivity to Double-Stranded DNA Breaks in Senescent Budding Yeast
Source: PLoS One. 2009 Dec 14;4(12):e8224. doi: 10.1371/journal.pone.0008224 (PMC2790616; doi:10.1371/journal.pone.0008224)
Supplement: Table S1 — Strains used in this study (0.09 MB DOC) [file pone.0008224.s007.doc]

| Strain | Genotype | Source |
| --- | --- | --- |
| AGY628 | *ho,hml::ADE1,mata*::hisG*, hmr::ADE, ade1, lys5, trp1*::hisG*, ade3::GAL-HO, ura3-52, LEU2*, intronless *ACT1* | [5] |
| AGY673 | AGY628 *URA3*::ai::HO cut site | [5] |
| STY1667 | AGY673 *tlc1*::*TRP1* | This study |
| STY95 | YPH501 *tlc1*::*LEU2*/*TLC1* | [1] |
| STY107 | YPH499 *tlc1*::*LEU2* | [1] |
| STY106 | YPH500 *tlc1*::*LEU2* | [1] |
| STY1609 | YPH499 *pol2-4* | This study |
| STY1610 | YPH499 *dnl4*::KanMX4 | This study |
| STY1715 | YPH499 *dnl4*::KanMX4 *tlc1*::*LEU2* | This study |
| STY1883 | YPH499 *rad59*::*HIS3* | This study |
| STY1882 | YPH499 *rad59*::*HIS3 tlc1*::*LEU2* | This study |
| STY1905 | YPH499 *rad50*::*HIS3* | [6] |
| STY1906 | YPH499 *rad50*::*HIS3 tlc1*::*LEU2* | [6] |
| STY1634 | YPH499 *mec1*::*HIS3* *smll*::KanMX4 | [7] |
| STY522 | YPH499 *tel1*::*HIS3* | [8] |
| STY1814 | YPH499 *tlc1*::*LEU2 smll*::KanMX4 | This study |
| STY1816 | YPH499 *mec1*::*HIS3* *smll*::KanMX4 *tlc1*::*LEU2* | This study |
| STY1817 | YPH499 *tel1*::*HIS3 tlc1*::*LEU2* | This study |
| STY1860 | YPH499 *smll*::KanMX4 | This study |
| STY479 | YPH499UT | [9] |
| STY1904 | YPH499UT *tlc1*::*LEU2* | [9] |
| STY1723 | YPH499 *rad52*::*URA3* *RAP1*-13Myc-KanMX6 pWJ1213 | This study |
| STY1725 | YPH499 *rad52*::*URA3* *RAP1*-13Myc-KanMX6 *tlc1*::*TRP1* pWJ1213 | This study |
| BY4742 | *MAT*α *his3*Δ*1 leu2*Δ*0 lys2*Δ*0 ura3*Δ*0* | [10] |
| BY4741 | *MAT*a *his3*Δ*leu2*Δ*0 met15*Δ*0 ura3*Δ*0* | [10] |
| STY1711 | BY4742 *tlc1*::*HIS3* | This study |
| STY1712 | BY4741 *tlc1*::*HIS3* | This study |
| STY1671 | BY4742 *chk1*::KanMX4 | Invitrogen |
| STY1791 | BY4742 *rad9*::KanMX4 | Invitrogen |
| STY1792 | BY4742 *rad24*::KanMX4 | Invitrogen |
| STY1829 | BY4742 *dun1*::KanMX4 | Invitrogen |
| STY1806 | BY4741 *mrc1*:: *HIS3* | This study |
| STY1713 | BY4742 *chk1*::KanMX4 *tlc1*::*HIS3* | This study |
| STY1852 | BY4742 *dun1*::KanMX4 *tlc1*::*HIS3* | This study |
| STY1853 | BY4742 *rad9*::KanMX4 *rad24*::KanMX4 *tlc1*::*HIS3* | This study |
| STY1854 | BY4742 *rad9*::KanMX4 *tlc1*::*HIS3* | This study |
| STY1855 | BY4742 *rad24*::KanMX4 *tlc1*::*HIS3* | This study |
| STY1856 | BY4742 *mrc1*:: *HIS3 tlc1*::*LEU2* | This study |
| STY1837 | BY4741 *pds1*::*LEU2* | This study |
| STY1851 | BY4742 *pds1*::*LEU2* *tlc1*::*HIS3* | This study |
