# Supplementary material for: Recruitment of Rad51 and Rad52 to Short Telomeres Triggers a Mec1-Mediated Hypersensitivity to Double-Stranded DNA Breaks in Senescent Budding Yeast
Source: PLoS One. 2009 Dec 14;4(12):e8224. doi: 10.1371/journal.pone.0008224 (PMC2790616; doi:10.1371/journal.pone.0008224)
Supplement: Table S2 — Oligonucleotide primers (0.04 MB DOC) [file pone.0008224.s008.doc]

| **Primer** | **Sequence (5’🡪3’)** | **Function** |
| --- | --- | --- |
| *TLC1*-5’ | CTGGTTCTGGTGGCATCTAT | To amplify the *tlc1*:*TRP1* fragment |
| *TLC1*-3’ | TTAAGCAGAGCACCCACATG | To amplify the *tlc1*:*TRP1* fragment |
| QL1 | CAGGCGGCGGAAGAAGTAA | To amplify the HOL fragment for ChIP |
| QL2 | GAGTGGTTGAGCTCGAGCAAT | To amplify the HOL fragment for ChIP |
| *ASP3*-5’ | TCACAGCAGAAGAACAGCAGATG | To amplify the *ASP3* fragment for ChIP |
| *ASP3*-3 | CGTAGCCGGAGTACAAACATAATTG | To amplify the *ASP3* fragment for ChIP |
| RAG513 | ATGTTCTAGCGCTTGCACCATC | To amplify the HO cut site |
| RAG515 | GGAGTTCAATGCGTCCATC | To amplify the HO cut site |
| *DDI2*-941 | AGCTTGTATTTAATGCGACAGG | To amplify the *DDI2* fragment for ChIP |
| *DDI2*-1128 | GTATCGATATGAATCAGATCGG | To amplify the *DDI2* fragment for ChIP |
| VIIL-968 | GGAAGCATATTTGAGAAGATG | To amplify the VIIL telomeric region for ChIP |
| VIIL-R | CTTGAAGCTCTAATTTGTGAGGATATCC | To amplify the VIIL telomeric region for ChIP |
| *DNL4*-5’ | GCGCAATCTGAGGAATCAGAAGACG | To amplify the *dnl4*::KanMX4 fragment |
| *DNL4*-3’ | CGGTTCCGGGTGTTCCATTAATCC | To amplify the *dnl4*::KanMX4 fragment |
| *POL2* S | GTAGAAGCGCCACTTCATCG | For *pol2-4* sequencing |
| *mrc1-HIS3-*5’ | ATGGATGATGCCTTGCATGCTTTGTCCTCGTTGACTG  CAAACTCTTGGCCTCCTCTAG | To amplify the *mrc1*::*HIS3* fragment |
| *mrc1-HIS3*-3’ | CTAATTATCAAAGCTATCTTGTCCGCTTTCAAAAAGT  TTAGAACACCTTTGGTGGAGG | To amplify the *mrc1*::*HIS3* fragment |
| *RAD59-HIS3-*5’ | CAAGCGAAGCCCAGTTCGAGCATATCGTATGATTCGA  CTACATACGGCACTCGTTCAGAATGACACG | To amplify the *rad59*::*HIS3* fragment |
| *RAD59-HIS3-*3’ | GCGTGCCTTTAGCATCCTCCAATTTGATAAAAGTCGG  CTTGCTATTAGTCGCTGACCTCTTGGCCTCCTCTAG | To amplify the *rad59*::*HIS3* fragment |
