# Supplementary material for: Recruitment of Rad51 and Rad52 to Short Telomeres Triggers a Mec1-Mediated Hypersensitivity to Double-Stranded DNA Breaks in Senescent Budding Yeast
Source: PLoS One. 2009 Dec 14;4(12):e8224. doi: 10.1371/journal.pone.0008224 (PMC2790616; doi:10.1371/journal.pone.0008224)
Supplement: Supporting Information References S1 — (0.03 MB DOC) [file pone.0008224.s009.doc]

**REFERENCES**

1. Teng SC, Zakian VA (1999) Telomere-telomere recombination is an efficient bypass pathway for telomere maintenance in Saccharomyces cerevisiae. Mol Cell Biol 19: 8083-8093.

2. Morrison A, Bell JB, Kunkel TA, Sugino A (1991) Eukaryotic DNA polymerase amino acid sequence required for 3'----5' exonuclease activity. Proc Natl Acad Sci U S A 88: 9473-9477.

3. Morrison A, Johnson AL, Johnston LH, Sugino A (1993) Pathway correcting DNA replication errors in Saccharomyces cerevisiae. EMBO J 12: 1467-1473.

4. Lea DEaC, C.A. (1948) The distribution of the numbers of mutants in bacterial populations. J Genet 49: 264-284.

5. Yu X, Gabriel A (1999) Patching broken chromosomes with extranuclear cellular DNA. Mol Cell 4: 873-881.

6. Teng SC, Chang J, McCowan B, Zakian VA (2000) Telomerase-independent lengthening of yeast telomeres occurs by an abrupt Rad50p-dependent, Rif-inhibited recombinational process. Mol Cell 6: 947-952.

7. Tseng S-F, Lin J-J, Teng S-C (2006) The telomerase-recruitment domain of the telomere binding protein Cdc13 is regulated by Mec1p/Tel1p-dependent phosphorylation. Nucl Acids Res 34: 6327-6336.

8. Tsai YL, Tseng SF, Chang SH, Lin CC, Teng SC (2002) Involvement of replicative polymerases, Tel1p, Mec1p, Cdc13p, and the Ku complex in telomere-telomere recombination. Mol Cell Biol 22: 5679-5687.

9. Taggart AK, Teng SC, Zakian VA (2002) Est1p as a cell cycle-regulated activator of telomere-bound telomerase. Science 297: 1023-1026.

10. Brachmann CB, Davies A, Cost GJ, Caputo E, Li J, et al. (1998) Designer deletion strains derived from Saccharomyces cerevisiae S288C: a useful set of strains and plasmids for PCR-mediated gene disruption and other applications. Yeast 14: 115-132.
